# Man-In-The-Middle Attacks in Vehicular Ad-Hoc Networks: Evaluating the Impact of Attackers’ Strategies

**DOI:** 10.3390/s18114040

**Published:** 2018-11-20

**Authors:** Farhan Ahmad, Asma Adnane, Virginia N. L. Franqueira, Fatih Kurugollu, Lu Liu

**Affiliations:** 1 Cyber Security Research Group, College of Engineering and Technology, University of Derby, Derby DE22 3AW, UK; v.franqueira@derby.ac.uk (V.N.L.F.); f.kurugollu@derby.ac.uk (F.K.); 2 Department of Computer Science, Loughborough University, Loughborough LE11 3TU, UK; 3 College of Engineering and Technology, University of Derby, Derby DE22 3AW, UK; l.liu@derby.ac.uk

**Keywords:** Vehicular Ad-Hoc network, security, Man-In-The-Middle Attack, smart cities, simulation, Intelligent Transportation System, Internet-of-Things

## Abstract

Vehicular Ad-Hoc Network (VANET), a vital component of Intelligent Transportation Systems (ITS) technology, relies on communication between dynamically connected vehicles and static Road Side Units (RSU) to offer various applications (e.g., collision avoidance alerts, steep-curve warnings and infotainment). VANET has a massive potential to improve traffic efficiency, and road safety by exchanging critical information between nodes (vehicles and RSU), thus reducing the likelihood of traffic accidents. However, this communication between nodes is subject to a variety of attacks, such as Man-In-The-Middle (MITM) attacks which represent a major risk in VANET. It happens when a malicious node intercepts or tampers with messages exchanged between legitimate nodes. In this paper, we studied the impact on network performance of different strategies which attackers can adopt to launch MITM attacks in VANET, such as fleet or random strategies. In particular, we focus on three goals of MITM attacks—message delayed, message dropped and message tampered. The simulation results indicate that these attacks have a severe influence on the legitimate nodes in VANET as the network experience high number of compromised messages, high end-to-end delays and preeminent packet losses.

## 1. Introduction

### 1.1. Motivation

**Figure 1:**
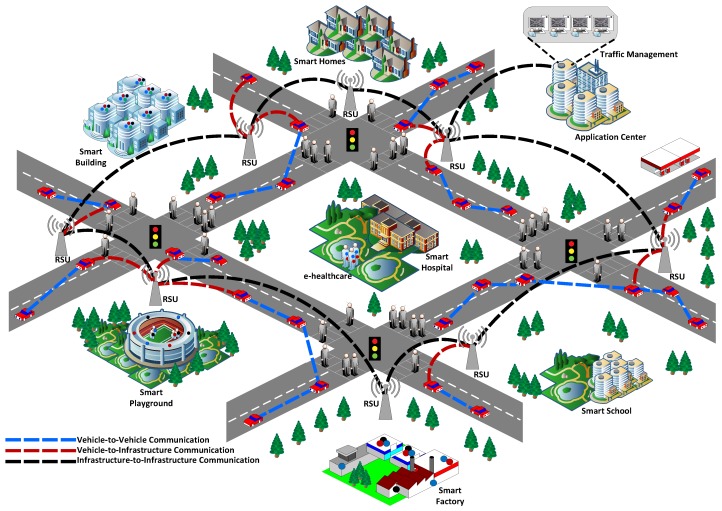
Illustration of VANET in smart cities.

Recently, Internet-of-Things (IoT) has emerged as a novel computing paradigm which provides connectivity to plethora of devices with sensing and communication capabilities to the Internet. These devices are connected to the Internet via various communication technologies including WiFi, ZigBee, Bluetooth, Long Term Evolution (LTE), 5G to provide a wide range of applications such as smart homes, smart grid, smart health, smart factories etc. [1,2,3]. Vehicular Ad-Hoc Network (VANET), which provides connectivity to millions of vehicles across the globe with the aim to improve traffic efficiency, is an important application of IoT and smart cities. In VANET, smart vehicles equipped with various sensors intelligently exchange messages with each other by virtue of vehicle-to-vehicle (V2V) or vehicle-to-infrastructure (V2I) communication to offer various applications including traffic management, black-ice and steep-curve warnings, congestion and traffic accident avoidance. Figure 1 illustrates the integration of VANET in smart cities where traffic safety is achieved by connecting vehicles to each other by via V2V and V2I communication.

Due to sensitive nature of VANET, a secure, trustworthy and attack-free environment is mandatory for disseminating messages throughout the network. Since, very critical information (such as black-ice warnings) is involved in VANET, thus it is paramount that this information is authentic and is generated from legitimate vehicles. Further, network should be capable of satisfying traditional security requirements, i.e., availability, authenticity, privacy, confidentiality, non-repudiation and integrity [4,5,6,7]. However, ensuring security in VANET is extremely challenging due to its susceptibility to different attacks such as Man-In-The-Middle (MITM) attacks which pose a severe risk in VANET [8,9]. In these attacks, malicious node (MITM) either eavesdrop or alter the messages exchanged between two legitimate vehicles. The exchanged information may contain sensitive and delay-intolerant information such as steep-curve warning. This results in the dissemination of compromised and incorrect information throughout the network, thus violating main pillars of security requirement, i.e., availability, confidentiality and integrity. Since, human life is directly involved in VANET, propagation of compromised information via malicious MITM nodes can have severe impact in the network. For instance, malicious nodes tampering steep-curve warning can result in traffic accidents.

MITM attacks are considered as severe attacks in VANET where malicious nodes have the ability to alter, drop or delay useful information in the network [10]. Adversaries can launch MITM attacks in two modes, i.e., passively or actively. Passively, the attacker can eavesdrop on the communication channel silently between legitimate vehicles. For instance, MITM attacker intercepts the communication channel of law-enforcement vehicles and share the communication with the interested organization for their own benefits. Actively, the attacker can drop, delay or change the content of received information in the network. For example, the attacker receives sensitive information such as a message about a traffic accident. If the attacker either changes the content of the message, or delay or drop the message, then this behavior of the MITM attacker can have severe impact on the network as the legitimate nodes will either receive compromised messages, or the information is delivered with high delays, or vehicles are prohibited from legitimate information in extreme cases.

Moreover, attacker pattern and strategies have significant impact on the performance of the network. As an illustration, the attacks launched by the attackers individually have different impact to the attacks launched in a collaborative manner. Thus, the performance of the network vary in both cases. However, MITM attacks with different attack strategies is ignored in the current literature. Therefore, we fill this research gap by proposing a study where we considered two patterns of the MITM attackers and study the impact of such attackers in VANET.

### 1.2. Research Question

Since, human life is directly involved in VANET, therefore, it must be paramount that only authentic and accurate information is disseminated throughout the network. MITM attackers pose severe risk to the network as they have the ability to compromise this authentic information. This results in a research question that what is the overall impact of these MITM attacks in a sensitive network like VANET? Further, how the network will behave for different strategies of MITM attacker? For instance, the impact caused by the MITM attacker with the capability to alter the sensitive messages will be different to the attack where the MITM attacker delays or drops the sensitive messages. Therefore, it is vital to study such attackers in VANET due to involvement of very sensitive messages (e.g., accident, steep-curve, black-ice warnings etc.) in the network.

### 1.3. Contributions

The major contributions of our research study are:

1. We identified various flavors of MITM attackers in VANET with different capabilities such as message tampering, message delaying and message dropping.
2. We established different strategies of the MITM attacker based on the mobility and distribution of the malicious nodes in VANET, and
3. We developed a simulation-based model to evaluate the impact of various MITM attacks under different attacker patterns.

The remaining of the paper is organized as follows. Section 2 is dedicated to the related work on MITM attacks in VANET. Section 3 elaborates different forms of MITM attacks in VANET. Simulation setup is discussed in Section 4 while results are explained in Section 5. Section 6 analyze the conclusions drawn from the simulation results.

## 2. Related Work

### 2.1. Man-In-The-Middle Attacks for Wired and Wireless Networks

Man-In-The-Middle (MITM) attacks are well known attacks in computer security where various surveys are conducted on MITM attack especially on wired and wireless networks. Stricot-Tarboton et al. [11] and Chen et al. [12] presented MITM attacks on wired networks. Stricot-Tarboton et al. presented a taxonomy of MITM on Hypertext Transfer Protocol Secure (HTTPS) by classifying the attackers into 4 tiers, i.e., state, target, behavior and vulnerability. On the other hand, Chen et al. focused on mathematical model for MITM attacks on Secure-Sockets Layer (SSL) protocols of wired networks. Unlike above studies, Conti et al. [13] presented a detailed survey covering MITM attacks on Open Systems Interconnection (OSI) layers of two mobile communication technologies, i.e., Global System for Mobile communications (GSM) and Universal Mobile Telecommunications System (UMTS), where MITM attacks were mapped according to their respective OSI layer. Furthermore, Glass et al. studied the impact of MITM attacks at the Medium Access Control (MAC) layer for wireless mesh and mobile ad-hoc networks [14]. The authors exploited the positive acknowledge property of the network to expose and identify malicious nodes performing MITM attack, thus achieving high detection rates and no false positives. Similarly, in [15], Kaplanis studied MITM attacks over WiFi networks. The author concluded that the absence of encryption over data link layer provides ideal platform for attackers to launch MITM attack. Further, a solution is provided by the author to tackle MITM attack over the WiFi network. In general, MITM attacks are studied for both wired and wireless technologies.

### 2.2. Man-In-The-Middle Attacks for VANET

Recently, a significant research effort is being carried out to achieve security and privacy in VANET. Raya et al. [16] were the pioneers to explore this area where they highlighted several security challenges in VANET. For instance, they argued that privacy preservation of vehicular users during highly mobile environment is extremely important. Fuentes et al. [17] presented various security requirements in their study, i.e., authentication, integrity, availability, confidentiality, and non-repudiation to solve above issues. On the other hand, different studies identified attacker models which were classified into two classes: high and low level [18,19]. However, these models are limited in scope and does not consider different strategies of these attackers. Similarly, several attacks were identified and classified according to security requirements [20,21,22,23,24]. For instance, DoS, MITM and eavesdropping attacks were major identified attacks. On the other hand, there are some studies which focused on performing risk assessment of various risks in VANET. For instance, Ahmad et al. provided a context-based risk assessment framework where various attacks along with their risk in VANET are identified [10]. Further, the attacks are categorized according to three classes, i.e., critical, major and minor. According to this study, MITM attacks are critical and have severe impact on VANET.

Afdhal et al. analyzed the impact of black hole attacks on two routing protocols Ad Hoc On-Demand Distance Vector (AODV) and Ad Hoc On-Demand Multipath Distance Vector (AOMDV) in the context of VANET [25]. The findings showed that MITM attacker performing black hole attack affects the performance of the routing protocols in terms of throughput. Further, Dhyani et al. proposed a solution to cater black holes by broadcasting unicast packets and integrating trust within AOMDV [26]. However, how the routing protocols will behave for urban and rural areas are missing from these studies. Grimaldo et al. studied the performance of wide range of routing protocols in VANET under black hole attacks [27]. The authors evaluated AODV, Optimized Link State Routing (OLSR), Dynamic Source Routing (DSR) and Destination-Sequenced Distance-Vector (DSDV) protocols for MITM attackers performing black hole attack for realistic network, concluding that the black hole attacks have severe impact on successful packet delivery in the network. Further, Purohit et al. studied the affect of black hole attacks for different routing protocols in VANET including AODV and Zone Routing Protocol (ZRP) [28]. However, the above studies consider routing protocols which are used mainly for traditional Mobile Ad-hoc Networks (MANET) environment and these routing protocols can not be used in VANET due to large-scale and high mobility of the vehicles. Almutairi et al. adopted a different approach to mitigate the impact of black-hole attacks in VANET where every vehicle maintains a trust routing table for neighbors [29]. Further, in case no neighbor is present near the legitimate vehicle, then the messages are exchanged with RSU to avoid any miscommunication within the network. However, there are three main drawbacks of this study. Firstly, this solution requires the presence of RSU for black-hole detection, which cannot be ensured in every scenario of VANET. Secondly, this solution always assume that the vehicles have low mobility. Thirdly, how this approach will behave in a rural scenario is not addressed by the authors. To identify MITM attacks with dropping ability, Cherkaoui et al. presented a study using quality control charts where abnormal activity is identified by continuously monitoring the network [30]. In the proposed method, two limits are identified for packet loses using statistical process control. This is an efficient technique to identify MITM attacks performing black-hole as the network is continuously monitored in a real-time environment.

Rawat et al. studied the data falsification attack in VANET where the MITM attacker propagates false information throughout the network [31]. The authors recommended the use of hash and contention window to avoid spreading the false information throughout the network. However, this study only focused on one aspect of MITM attacker. Further, Leinmuller et al. identified various message forging attacks in VANET [32]. Moreover, the authors modeled and equipped the attacker along the RSU with message forging capability and studied its effect on the network. The main drawback of this study is that the attacker is always stationary which is not realistic in real VANET environment. On the other hand, Grover et al. studied different position forging attacks in VANET with the focus on creation of illusion of non-existent event in the network [33]. The authors studied the impact of position forging attacks based on the vehicle speed, channel utilization and total collisions in the network. Further, a solution was proposed by the authors which utilizes RSU to identify attackers performing position forging attacks. However, there are two drawbacks of this study. First, the solution relies on RSU which may not be present in every scenario in VANET, and secondly, the authors did not mention about the distribution pattern of the attacker nodes in the network.

To sum up, MITM attacks are severe in VANET and there is no direct study, which provides a detailed comparison of different types of MITM attacks. We fill this gap by proposing a simulation-based study, where we have implemented and evaluated three different types of MITM attacks in VANET. Table 1 provides a high-level qualitative comparison of different studies on MITM attacks in VANET.

## 3. Man-In-The-Middle Attacks

**Figure 2:**
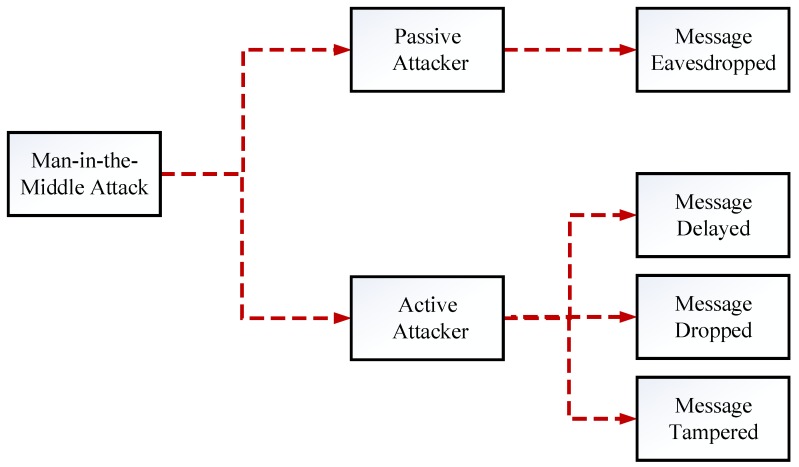
Man-In-The-Middle Attacks in VANET.

The term “*Man-In-The-Middle*” has been derived from basketball scenario where a player in the middle tries to intercept the ball while other two players try to pass it [34]. The same concept is derived in VANET, where MITM attacker jeopardize communication and modify messages among legitimate vehicles. Such attacks leave severe consequences on the network, especially, if content of message contains safety related information. In VANET, the attacker must satisfy following two conditions in order to implement MITM attack, i.e., (1) Firstly, the message containing significant information must be received by the attacker node, and (2) Secondly, the attacker must be able to interpret the content of message [35]. MITM attacks in VANET can be launched under following two modes as depicted in Figure 2.

1. *Passive Mode:* Passively, attacker can eavesdrop on the communication channel between legitimate vehicles, e.g., police vehicles or ambulances.
2. *Active Mode:* Actively, attacker can drop, delay or change the content of received information in the network.

When an event occurs in VANET, the transmitted packet ($M_{G}$) from message generated vehicle contains at least following three important information, i.e., data regarding the event, location of the event generation, and time of the event occurrence along with extra information such as protocol versions, message IDs etc. [36,37]. For the sake of clarity and relevance, the message generated from the vehicle will contain following three information in our study, i.e., data, location and time.

(1) $$M_{G}=\left\{data,location,time\right\}$$

where, data contains the information generated by the vehicle which can be either related to safety or non-safety application. Location contains the coordinates of the event generation vehicle in terms of *x*, *y*, *z*, which can be achieved via GPS of the vehicle. Further time represents the message generation time $\left(t_{send}\right)$. Therefore, above equation can be further expended as:

(2) $$M_{G}=\left\{safety\;data,non\;safety\;data,loc_{x},loc_{y},loc_{z},t_{send}\right\}$$

In presence of malicious nodes in the network performing MITM attack, the attacker can compromise the content of the Equation (1) in terms of data, location or time. Compromising the data containing safety information can create disaster in the network. Similarly, tampering the location of the message sender can confuse the legitimate vehicle about the message receiver. Further, changing the time of the message can also have impact on the network as the receiver might ignore the message which is out-dated or very old. In light of these characteristics, we consider an attacker, which can tamper the “legitimate data” and propagates the “compromised data” throughout the network. Actively, attacker can launch MITM in following three manners.

1. Delay the legitimate message
2. Drop the legitimate message
3. Tamper the legitimate message

**Figure 3:**
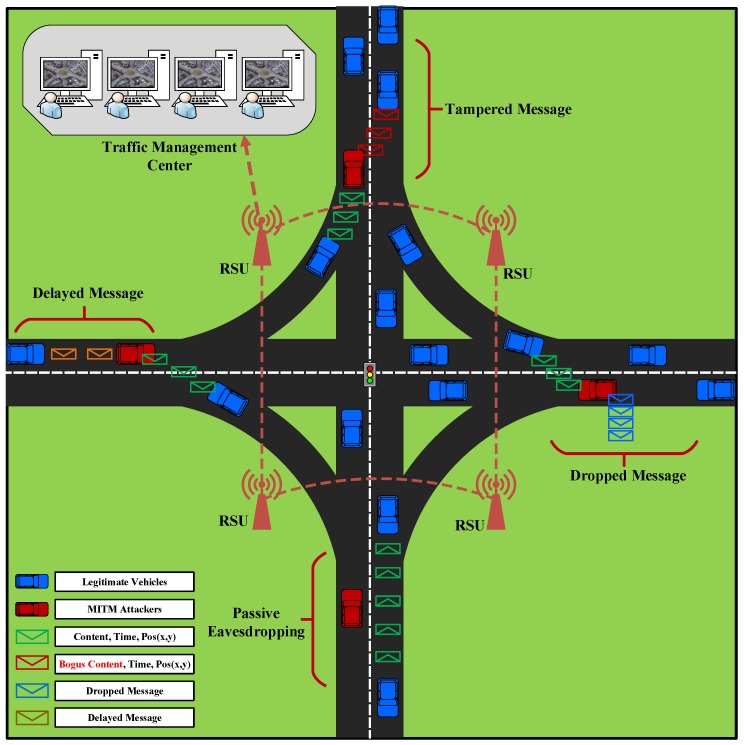
Illustration of Passive and Active MITM Attacks in VANET.

Figure 3 depicts the illustration of both passive and active MITM attacks in VANET. It can be seen that passively, MITM attacker can eavesdrop on the communication between legitimate vehicles and actively, the MITM attacker can drop, delay or alter the authentic messages which are propagated throughout the network.

### 3.1. MITM as Message Delayed

The success of VANET relies on the successful transmission of messages to every legitimate vehicle. In this attack model, the malicious nodes deliberately delay the messages, i.e., the messages are forwarded to neighbour nodes with a factor of ‘delay’. Due to sensitive nature of the messages in VANET, delaying such messages can create disaster in the network. For instance, consider a scenario where legitimate vehicles are sharing information about steep-curve during night. Delaying such message by a malicious node can result in extreme situation, where, the legitimate vehicles are unable to receive this messages on time. Therefore, such vehicles have to take step in real-time to avoid an accident scenario. Further, this situation can also put the human life in danger.

### 3.2. MITM as Message Dropped

This type of attack also refers to “black hole” attacks in VANET, where attacker intentionally drops the received legitimate message $(M_{G})$, thus suppressing the further propagation of $M_{G}$ [38]. Hence, this action of the attacker prohibits the legitimate vehicles to receive any kind of message (safety and non-safety) as the messages never reaches to their destination. Dropping the safety-related messages can have significant impact on the network as it contains sensitive information such as collision avoidance. As an illustration, consider a scenario where legitimate vehicles are broadcasting the messages regarding black-ice on the road. Dropping such information can put the life of vehicular users in danger as they are prohibited from receiving sensitive information by the attacker.

### 3.3. MITM as Message Tampered

In this attack, the attacker particularly targets the content of the received message. Whenever a message is received at the malicious node, attacker changes the content of the message. This form of attack has severe impact on the network as the content may contain sensitive information. For instance, legitimate vehicle broadcast a message during heavy rain that “there is steep-curve ahead, slow down”. The message is received at the attacker node, where the content is intentionally altered to “there is no steep-curve, speed up”. This message is misleading for the legitimate vehicles and it can create disaster (such as accident occurrence) in the network.

Further, every transmitted message in VANET contains three important information, i.e., (1) data, (2) time, and (3) location. In this attack, the attacker have the ability to change “data”, “transmission time” or “transmission location”. In this particular attack, the attacker can change,

- “data” into misleading “compromised data”
- “transmission time” into compromised transmission time by changing it with garbage time $t_{g}$
- coordinates of sender location (loc(x,y,z)) into unknown location $\left(loc\left(x_{a},x_{b},x_{c}\right)\right)$

In this paper, we implemented the above three versions (message delay, message drop, message alter) of the MITM attack. It is worth mentioning that MITM nodes can violate integrity, authentication, confidentiality and availability security requirement for VANET. Further, to evaluate the impact caused by these attacks, we considered two different strategies of attackers performing MITM.

1. First, the attackers are distributed randomly across the network
2. Second, the attackers exist in fleet structure where the attacks are launched in a collaborative manner.

**Figure 4:**
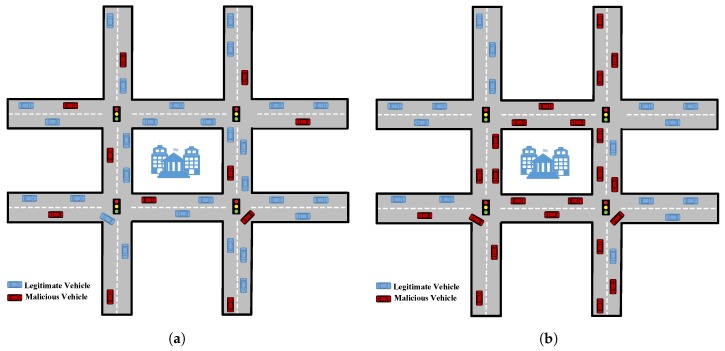
Attacker Pattern (**a**) Distributed (**b**) Fleet.

Figure 4 highlights the difference of attacker pattern in the network. In the next section, we describe the simulation environment for these MITM attacks in VANET.

## 4. Simulation Environment

### 4.1. Simulation Setup

**Figure 5:**
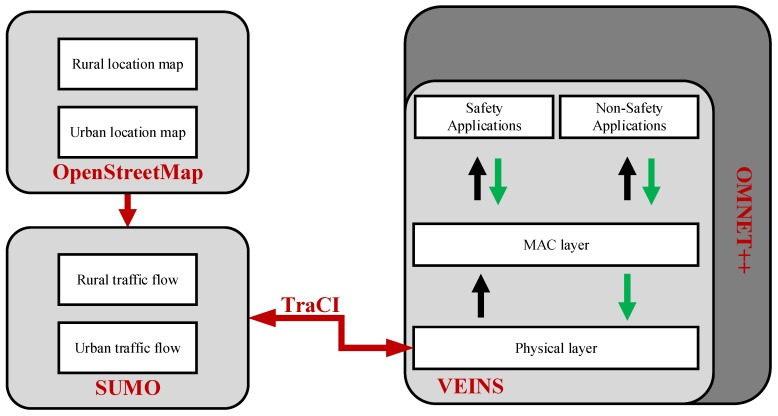
Workflow of Veins Simulation Framework.

The core objective of our simulation is to study performance of the vehicular networks in presence of malicious nodes performing MITM attacks. To facilitate our simulations, we used Veins, which is an open source framework and is used widely for simulations of vehicular networks [39,40]. Veins is built on top of two popular simulators: SUMO (traffic simulator) [41] and OMNET++ (discrete event simulator) [42] as depicted in Figure 5. SUMO provides traffic patterns for specific realistic map while OMNET++ provides various modules (application layer, DSRC and PHY layer) to ensure realistic network behavior. A small patch “TraCI” is used for communication between OMNET++ and SUMO [43]. Whenever, an event (accident information) is triggered in OMNET++, TraCI enables the vehicles in SUMO to change their route by sending out respective commands.

1. **Scenario 1:** Attackers are distributed randomly across the network, and
2. **Scenario 2:** Attackers are present together in a fleet structure.

### 4.2. Simulation Scenario Setup

**Figure 6:**
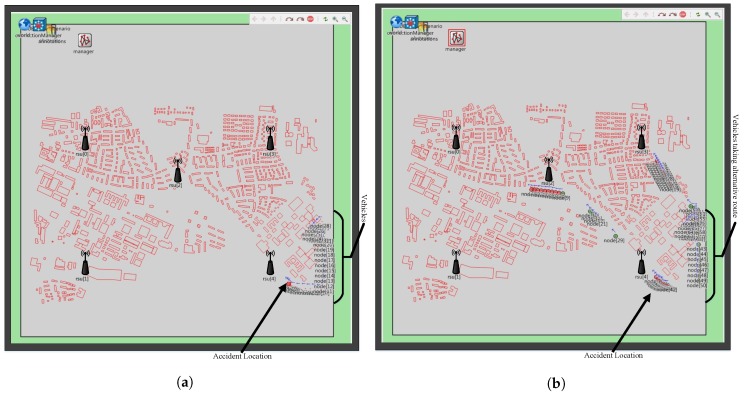
Snapshot of Veins (**a**) Vehicles during accident (**b**) Vehicles after accident.

To evaluate MITM attacks in VANET, we used the default map in Veins as depicted in Figure 6a. We introduced 100 vehicles in the network which are enough for many urban scenarios [44]. When vehicles are in the network, an event (accident) is generated at a random location. The vehicles share this information with its neighbors by broadcasting it. Vehicles upon receiving this information waits for sometime and diverts to other routes to avoid congestion as depicted in Figure 6b. We then injected 10%, 20%, 30%, 40% and 50% malicious nodes in the network respectively to study the impact caused by such attackers, where, the attackers delays, drops or tamper the shared messages. To study the attacker pattern, we created two scenarios:

In scenario 2, we polluted network with ‘*N*’ malicious nodes in such a way that they obliged specific pattern in the network, i.e., N/3 MITM nodes exist near the event occurrence, N/3 in the center of the network, while the rest of the MITM nodes (N/3) are located at the end of the network. Different parameters to perform simulations is described in Table 2.

### 4.3. Performance Evaluation Metrics

In order to evaluate the performance of VANET in presence of attackers, we implemented following evaluation criteria which can evaluate the MITM attacks in VANET. These are:

- **End-to-End Delay:** This metric is related to QoS of the network, indicating the delay caused to packet generated by legitimate node to be shared with neighbouring nodes. E2ED is the difference of packet generation time $\left(T_{G}\right)$ and packet reception time $\left(T_{R}\right)$ which is calculated as follows:
  (3) $$E2ED=T_{R}-T_{G}$$
- **Content Delivery Ratio (CDR):** Content delivery ratio shows the amount of messages which are received successfully by the legitimate vehicles [45]. Let $M_{R}$ are the number of received messages and $M_{PRE}$ are the number of messages which are expected to be received within the network, then CDR is given as:
  (4) $$CDR=\frac{M_{R}}{M_{PRE}}$$
  Let ‘*N*’ is the total number of vehicles which are transmitting ‘$M_{TRANS}$’ messages, then $M_{PRE}$ is calculated as:
  (5) $$M_{PRE}=N\times M_{TRANS}$$
- **Packet Loss Ratio (PLR):** Packet loss ratio shows the amount of the messages which are lost due to MITM nodes. Let $M_{T}$ are the total number of messages, out of which $M_{L}$ messages are lost, then PLR is given as:
  (6) $$PLR=\frac{M_{L}}{M_{T}}$$
  $M_{T}$ includes messages which are received at both legitimate and malicious nodes. Let $M_{R}$ is the number of received messages at legitimate nodes and $M_{L}$ is the amount of messages lost at the MITM nodes, then $M_{T}$ is given as:
  (7) $$M_{T}=M_{R}+M_{L}$$
- **Number of Compromised Messages:** This metric indicates the number of messages compromised (either tampered or delayed) from the malicious node.
- **Number of Dropped Messages:** This metric is defined for MITM which is dropping the messages received from legitimate nodes. This metric shows the amount of messages dropped by the attackers in the network.

## 5. Results and Discussion

In this section, we first provide the simulation results of three versions (message delayed, message dropped and message tamper) of MITM attacks in VANET. Then, we focused on the discussion of some the possible solutions to cater MITM attacks.

### 5.1. Simulation Results

This section is dedicated to discuss results of MITM attack in VANET. We simulated MITM attackers according to above two scenarios (distributed attackers and fleet of attackers) and evaluated network efficiency based on the evaluation metric defined above. Further, each simulation scenario is carried out twenty five times with random seed value to ensure unique initial vehicle assignment within the network every time. Moreover, the simulation results presented below are the average of twenty five runs for each simulation scenario.

#### 5.1.1. Message Delay Attacks

**Figure 7:**
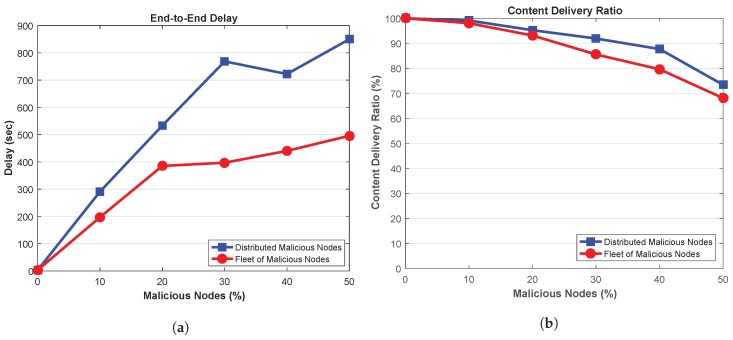
(**a**) End-to-End Delay (**b**) Content Delivery Ratio.

Figure 7a shows end-to-end (E2E) delay in the presence of MITM which are delaying the packets by 2 s. It can be seen that the E2E delay increases when the network is introduced with such malicious nodes which are delaying the legitimate messages. Ideally, the legitimate vehicles should receive such legitimate messages with minimum delay, however, MITM attackers with message delaying capability prohibits the legitimate nodes to receive the messages in time. Further, this figure also depicts that E2E delay increases when the attackers are distributed throughout the network. Since, a wide portion of the network is affected due to distributed attackers, therefore, the overall E2E delay increases in the network. On the other hand, attackers in fleet are only delaying the packets at certain locations. As a result, network experience low E2E delays in presence of attackers in fleet structure. Further, it can also be seen that for 10% malicious nodes, the network with distributed attackers achieve about 47.94% high E2E delays than the network containing fleet malicious nodes. This delay further increases to 73.44% when the network is injected with 50% malicious nodes.

Next, the amount of content delivered in the network is depicted in Figure 7b, showing that content can be delivered to the legitimate vehicles in presence of MITM attackers with delaying capabilities. This metric indicates that the messages arrived at the legitimate nodes but with certain delay. Further, high CDR is achieved in the network in presence of distributed malicious nodes, while, the network with fleet of malicious nodes attains low CDR. This is due to the fact that fleet of malicious vehicles are delaying the packets together, thus, high number of packets are delayed in such locations and as a result, the legitimate vehicles receives the content but not in time.

Figure 7b also suggests that for 10% malicious nodes, the network containing distributed malicious nodes achieve about 1.1% high CDR as compared to the network with attackers in fleet pattern. Moreover, the network with distributed malicious nodes achieves about 7.8% high CDR than the network with fleet attackers. Thus, fleet vehicles affect the network more by delivering less amount of content.

**Figure 8:**
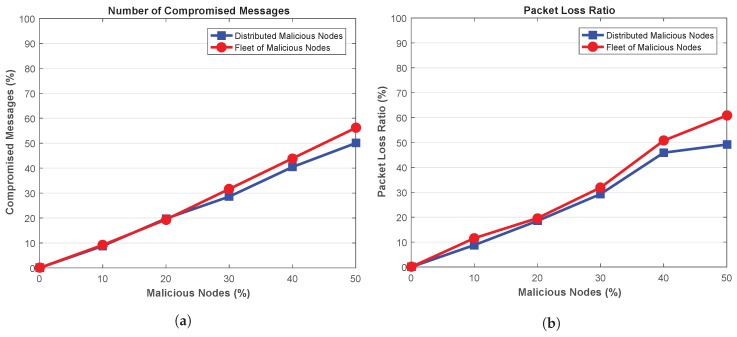
(**a**) Compromised Messages (**b**) Packet Loss Ratio.

Further to the above discussion, the number of compromised messages generated by the malicious nodes is depicted in Figure 8a. It can be seen that the number of compromised messages increases with the increase in malicious nodes in the network. However, high number of messages are compromised by the fleet malicious nodes than distributed malicious nodes. The attackers in fleet are working together to delay the messages, thus, higher number of messages are delayed and compromised. For instance, for a network containing 10% attackers, fleet malicious nodes compromises about 4.43% messages than distributed attackers. The compromised messages increases to 12.23% when the network is polluted with 50% attackers. This shows that high number of messages are compromised in presence of attackers in fleet structure as the attackers are compromising the messages in a collaborative manner.

Packet Loss Ratio (PLR) in presence of malicious nodes in depicted in Figure 8b. It shows that PLR increases with the increase in the malicious nodes in the network. However, the presence of fleet attackers deteriorates the network more as high number of packets are lost by such attackers. This is due to the fact that fleet attackers compromises high number of messages, therefore, the resulting network experience high packet loss. On the other hand, presence of distributed malicious nodes also results in packets loss, but, the resulting packet loss is less than the fleet attackers. For instance, when the network is flooded with 50% malicious nodes, about 23.75% more packets are lost in presence of fleet attackers as compared to distributed attackers.

#### 5.1.2. Message Drop Attacks

**Figure 9:**
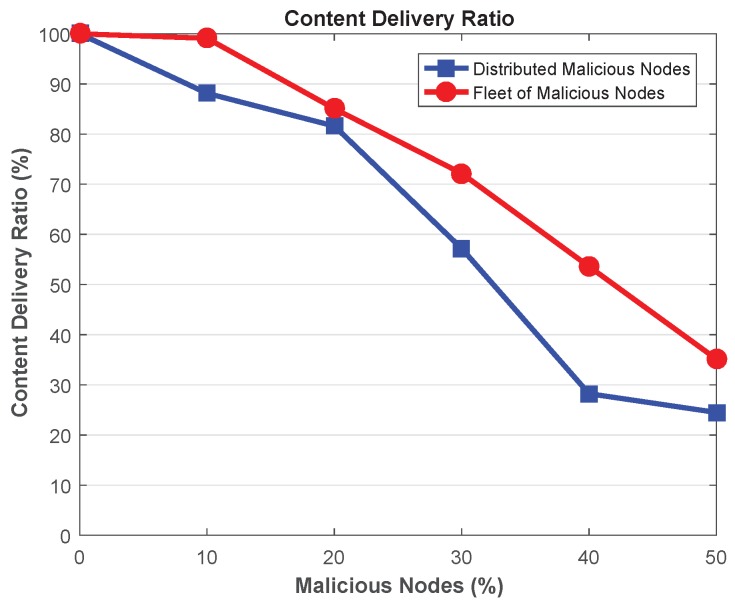
Content Delivery Ratio.

Content delivery ratio in case of message drop attacks is presented in Figure 9, depicting that CDR decreases with the introduction of malicious nodes in the network. Further, the network assures high number of content when it is polluted with fleet of malicious attackers. Since, the attack vector of such attacker is limited to specific location, therefore, content is lost only in that location. The nodes may be able to receive messages from other legitimate nodes within its neighbourhood. On the other hand, the scope of distributed malicious attackers is not limited to specific location, hence, low CDR is achieved in this scenario.

Further, as mentioned earlier, CDR decreases when the malicious nodes are introduced in the network. Specifically, low CDR is achieved in presence of distributed malicious nodes with the capability to drop legitimate packets. For instance, when the network contains 10% attackers, the network with fleet malicious nodes attain about 12.44% higher CDR than the network with distributed malicious nodes. However, by increasing the ratio of malicious nodes to 50%, network with fleet attackers achieves 44.89% high CDR than the distributed malicious nodes. This is due to the fact that significant amount of messages are dropped by distributed malicious vehicles as they are spread throughout the network. Therefore, content is always lost the vicinity of such attackers. On the other hand, in case of fleet attackers, content is only dropped in specific locations of the network, while, the network where attackers not present can share messages with their neighboring vehicles.

**Figure 10:**
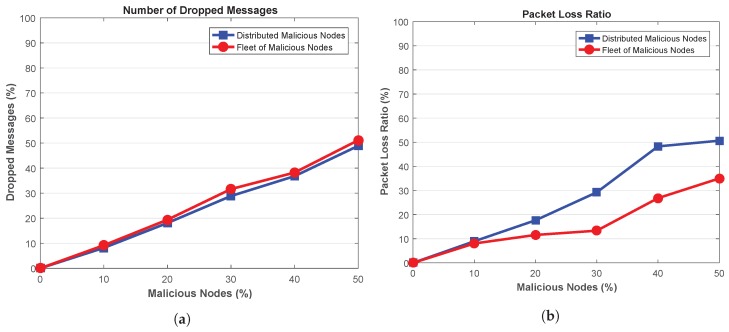
(**a**) Dropped Messages (**b**) Packet Loss Ratio.

Next, the number of dropped messages by malicious nodes is shown in Figure 10a, suggesting that high number of messages are dropped when the ratio of malicious nodes is increased in the network. Both attacker patterns (distributed and fleet) have high impact on the network in terms of the amount of messages dropped in the network, i.e., both distributed and fleet attackers drops almost similar number of messages. However, the network with fleet attackers achieve a little higher packet drop rate than distributed attackers due to their collaborative nature of attack launching. Increasing such attackers increases the drop rate of the messages in the network. For example, for 50% attackers in the network, fleet attackers drop 4.60% more packets than distributed attackers.

Though, almost both fleet and distributed attackers results in dropping the packets in the network, its impact can be elaborated more via packet loss ratio in Figure 10b. This shows that the network with distributed malicious nodes results in high number of lost packets. As the attack-vector of the distributed malicious nodes is exposed to wide area of the network, therefore, increasing such malicious nodes results in high PLR. For instance, for a network with 10% malicious nodes, network with distributed attackers experience about 11.31% high PLR than fleet attackers. This ratio increases to 44.67% when the network is flooded with 50% malicious nodes.

#### 5.1.3. Message Tamper Attacks

As described above, the malicious node can either alter data, time or location of the legitimate message. In this particular attack, we focused on the data of the message. Thus, whenever an attacker received a message, the content is tampered by this malicious nodes into garbage data which is then shared with the neighboring vehicles.

**Figure 11:**
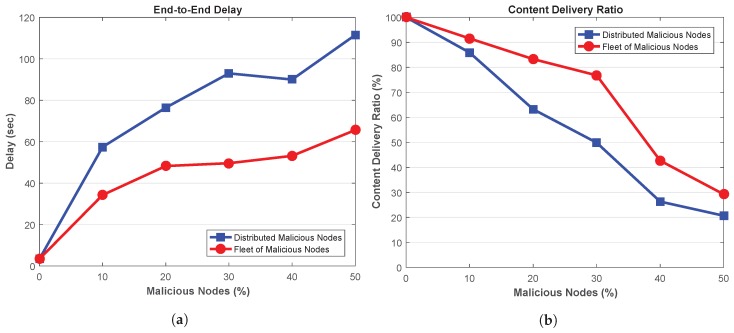
(**a**) End-to-End Delay (**b**) Content Delivery Ratio.

Figure 11a shows the end-to-end delay of the network in presence of the malicious nodes which are changing the content of the messages. First, it can be seen concluded that E2E delay of the network increases with the increase in malicious nodes. Second, high E2E delay is achieved by the network in presence of distributed malicious nodes due to their wide spread attack scope. On the other hand, fleet of malicious nodes are launching attacks which are limited to specific location, therefore, the low E2E delay is achieved as legitimate messages are shared in a large section of the network. As an illustration, for a network with 50% malicious nodes, network containing distributed attackers achieves 69.91% high E2E delays than fleet attackers.

The ability of the network to transmit legitimate messages within the network via CDR is depicted Figure 11b. It shows that CDR decreases when the number of malicious nodes increases within the network. As the malicious nodes are changing the content of the legitimate messages, therefore increasing such malicious nodes results in low values of CDR as the ratio of garbage data increases within the network. Further, the attack pattern also affects CDR. It can be seen that when the network contains attackers in fleet, high CDR is achieved as compared to distributed malicious nodes. This is due to the fact that fleet of attackers are located in particular section of the network, thus, only a portion of the network is affected by these attacks. In the meantime, legitimate messages are transmitted between the nodes in large section of the network, thus high CDR is achieved. On the other hand, due to wide-spread attack vector of the distributed malicious nodes, large section of the network is affected. As a result the network achieve low CDR values. Further, figure also depicts that for 50% malicious nodes, the network with fleet attackers achieves about 41.48% high CDR as compared to distributed attackers.

**Figure 12:**
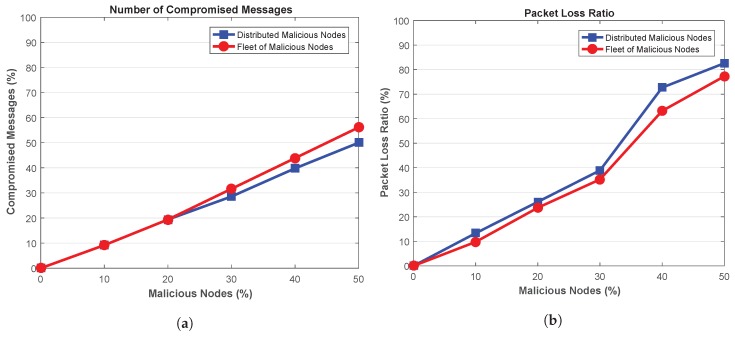
(**a**) Compromised Messages (**b**) Packet Loss Ratio.

Next, the number of compromised messages is shown in Figure 12a. It can be seen that the high number of messages are tampered and compromised in presence of high number of malicious nodes. Further, high number of messages in the network are in compromised state in presence of fleet of malicious nodes. As the attackers are launching attack together, therefore, network is affected more in presence of such attackers. For 50% malicious attackers, the network with fleet vehicles are compromising about 12.23% more packets than distributed attackers.

The impact of the attackers on the network in terms of lost packets in depicted in Figure 12b. Ideally, small value of PLR is desired. Figure shows that network is affected more when malicious nodes are increased within the network. Moreover, the attack pattern of the malicious nodes also results in different PLR. The distributed malicious nodes affect the network more as high PLR is achieved in the network. As mentioned earlier, these attacker have high impact on the network due to their wide-range of attack vector. As a result, high number of legitimate packets are lost within the network. On the other hand, fleet of attacker are only targeting at the specific portion of the network, thus, packets are lost in such location only. For 50% distributed attackers, the network experience about 6.89% more lost as compared to the network containing fleet attackers.

### 5.2. Discussion

The above sections clearly depicted that all the three flavors of MITM attacks have high impact on VANET, where the network achieves high E2E delays, low content delivery ratios, higher packet loses and the propagation of high number of compromised messages. It can be seen that the distributed malicious attackers affect the network in terms of E2E delays and CDR. The fleet MITM attackers, on the other hand, influence the VANET in terms of compromised messages and PLR. Therefore, in order to design a security solution in VANET, these characteristics of MITM should be taken into account before implementing in the real network. For instance, encrypting the messages can reduce the probability of disseminating false information among network entities within VANET. Further, trust-based solutions can also be employed within the network to cater MITM attacks with delaying and tampering abilities. Trust-based environment can enable the legitimate vehicles to identify and evaluate the trustworthiness of the received messages, thus reducing the probability of attackers to launch MITM attacks. Similarly, different plausibility checks in the network can also be helpful to reduce the propagation of compromised messages. These checks can be based on message threshold and detection ranges, message acceptance range, and some mobility controlled criteria including vehicles speed and direction.

## 6. Conclusions

VANET is the future of ITS where a secure and attack-free environment is required to achieve the desired traffic efficiency. However, due to the open nature of VANET, it is exposed to various attacks, such as MITM attacks.

This paper provided a detailed study of MITM attacks in VANET. We implemented three flavors of MITM attacks in VANET to study the impact caused by them. Results suggested that these attacks have a massive influence on the network in terms of low content delivery, high end-to-end delay, compromised messages and packet loses. Moreover, poor performance of the network is observed for the attacker model with fleet structure as compared to distributed attackers in terms of compromising the message content. The main reason for such poor performance is that fleet attackers jeopardize a significant amount of resources and communication channel in the network, creating a congestion in the network while distributed attackers only impact resources on a small scale.

As a future work, we will extend this research by evaluating the impact of the MITM attack models for various contexts of VANET based on the mobility of nodes. Further, our focus in the future will also be to design a trust-based solution to cater MITM attacks in VANET.

## Figures and Tables

**Table 1:** Comparison of Different Studies on MITM Attacks in VANET.

Studies	Man-In-The-Middle Attacks			Attacker Pattern	
	Message Tampered	Message Delayed	Message Dropped	Distributed Attackers	Fleet of Attackers
Afdhal et al. [25]			✓	✓	
Dhyani et al. [26]			✓	✓	
Grimaldo et al. [27]			✓	Unspecified	
Purohit et al. [28]			✓	✓	
Almutairi et al. [29]			✓	✓	
Cherkaoui et al. [30]			✓	✓	
Rawat et al. [31]	✓			✓	
Leinmuller et al. [32]	✓			Unspecified	
Grover et al. [33]	✓			Unspecified	
Proposed Study	✓	✓	✓	✓	✓

**Table 2:** Simulation Details.

Parameter		Value
**Simulation** **Framework**	Network Simulator	OMNET++ 5.0
	Traffic Simulator	SUMO 0.25.0
	V2X Simulator	VEINS 4.4
**Simulation** **Details**	No. of Vehicles	100
	No. of RSUs	5
	No. of Malicious Nodes	10%, 20%, 30%, 40%, 50%
	Simulation Area	2.5 km × 2.5 km
	Simulation Time	1000 s
	Accident Start time	75 s
	Accident Duration	50 s
	Communication Range	250 m
	Vehicle Maximum Speed	13.9 km/h
**Protocols**	MAC Protocol	IEEE 802.11p
	Network Protocol	IEEE 1609.4 (WAVE)
	Radio Propagation Model	Simple Path Loss
	Data Size	1024 bits
	Header Size	256 bits

## Abbreviations

The following abbreviations are used in this manuscript:

AODV	Ad Hoc On-Demand Distance Vector
AOMDV	Ad Hoc On-Demand Multipath Distance Vector
CDR	Content Delivery Ratio
DoS	Denial-of-service
DSR	Dynamic Source Routing
DSDV	Destination-Sequenced Distance-Vector
E2E	End-to-end
GSM	Global System for Mobile communications
HTTPS	Hypertext Transfer Protocol Secure
ITS	Intelligent Transportation System
IoT	Internet of Things
LTE	Long Term Evolution
MANET	Mobile Ad-hoc Networks
MITM	Man-In-The-Middle
MAC	Medium Access Control
OLSR	Optimized Link State Routing
OSI	Open Systems Interconnection
PLR	Packet Loss Ratio
RSU	Roadside Unit
SSL	Secure-Socket Layer
UMTS	Universal Mobile Telecommunications System
VANET	Vehicular Ad-Hoc Networks
WHO	World Health Organization
ZRP	Zone Routing Protocol

## References

[1] Yaqoob I., Hashem I.A.T., Mehmood Y., Gani A., Mokhtar S., Guizani S. (2017). Enabling Communication Technologies for Smart Cities. IEEE Commun. Mag..

[2] Chen S., Xu H., Liu D., Hu B., Wang H. (2014). A Vision of IoT: Applications, Challenges, and Opportunities With China Perspective. IEEE Internet Things J..

[3] Mehmood Y., Ahmad F., Yaqoob I., Adnane A., Imran M., Guizani S. (2017). Internet-of-Things Based Smart Cities: Recent Advances and Challenges. IEEE Commun. Mag..

[4] Zhong H., Huang B., Cui J., Xu Y., Liu L. (2018). Conditional Privacy-Preserving Authentication Using Registration List in Vehicular Ad Hoc Networks. IEEE Access.

[5] Sun Y., Wu L., Wu S., Li S., Zhang T., Zhang L., Xu J., Xiong Y. Security and Privacy in the Internet of Vehicles. Proceedings of the International Conference on Identification, Information, and Knowledge in the Internet of Things (IIKI).

[6] Lu Z., Qu G., Liu Z. (2018). A Survey on Recent Advances in Vehicular Network Security, Trust, and Privacy. IEEE Trans. Intell. Transp. Syst..

[7] Cui J., Xu W., Zhong H., Zhang J., Xu Y., Liu L. (2018). Privacy-Preserving Authentication Using a Double Pseudonym for Internet of Vehicles. Sensors.

[8] Al-kahtani M. Survey on Security Attacks in Vehicular Ad hoc Networks (VANETs). Proceedings of the 6th International Conference on Signal Processing and Communication Systems (ICSPCS).

[9] Ahmad F., Hall J., Adnane A., Franqueira V.N.L. Faith in Vehicles: A Set of Evaluation Criteria for Trust Management in Vehicular Ad-Hoc Network. Proceedings of the IEEE International Conference on Internet of Things (iThings) and IEEE Green Computing and Communications (GreenCom) and IEEE Cyber, Physical and Social Computing (CPSCom) and IEEE Smart Data (SmartData).

[10] Ahmad F., Adnane A. A Novel Context-based Risk Assessment Approach in Vehicular Networks. Proceedings of the IEEE 30th International Conference on Advanced Information Networking and Applications Workshops.

[11] Stricot-Tarboton S., Chaisiri S., Ko R.K.L. Taxonomy of Man-in-the-Middle Attacks on HTTPS. Proceedings of the 2016 IEEE Trustcom/BigDataSE/ISPA.

[12] Chen Z., Guo S., Duan R., Wang S. Security Analysis on Mutual Authentication against Man-in-the-Middle Attack. Proceedings of the First International Conference on Information Science and Engineering.

[13] Conti M., Dragoni N., Lesyk V. (2016). A Survey of Man In The Middle Attacks. IEEE Commun. Surv. Tutor..

[14] Glass S.M., Muthukkumarasamy V., Portmann M. Detecting Man-in-the-Middle and Wormhole Attacks in Wireless Mesh Networks. Proceedings of the International Conference on Advanced Information Networking and Applications.

[15] Kaplanis C. (2015). Detection and Prevention of Man in the Middle Attacks in Wi-Fi Technology. Master’s Thesis.

[16] Raya M., Papadimitratos P., Hubaux J.P. (2006). Securing Vehicular Communications. IEEE Wirel. Commun. Mag..

[17] de Fuentes J.M., Gonzalez-Tablas A.I., Ribagorda A., Cruz-Cunha M.M., Moreira F. (2010). Overview of Security Issues in Vehicular Ad-Hoc Networks. Handbook of Research on Mobility and Computing, Evolving Technologies and Ubiquitous Impact.

[18] Sumra I.A., Hasbullah H., Lail J., Rehman M. (2011). Trust and Trusted Computing in VANET. Comput. Sci. J..

[19] Papadimitratos P., Buttyan L., Holczer T., Schoch E., Freudiger J., Raya M., Ma Z., Kargl F., Kung A., Hubaux J.P. (2008). Secure Vehicular Communication Systems: Design and Architecture. IEEE Commun. Mag..

[20] Sakiz F., Sen S. (2017). A Survey of Attacks and Detection Mechanisms on Intelligent Transportation Systems: VANETs and IoV. Ad Hoc Netw..

[21] Vinh H.L., Cavalli A.R. (2014). Security Attacks and Solutions in Vehicular Ad Hoc Networks: A Survey. Int. J. AdHoc Netw. Syst..

[22] Siddiqui N., Khaliq K.A., Pannek J. VANET Security Analysis on the Basis of Attacks in Authentication. Proceedings of the 5th International Conference on Dynamics in Logistics.

[23] Ahmad F., Adnane A., Franqueira V.N.L. (2016). A Systematic Approach for Cyber Security in Vehicular Networks. J. Comput. Commun..

[24] Hamida E.B., Noura H., Znaidi W. (2015). Security of Cooperative Intelligent Transport Systems: Standards, Threats Analysis and Cryptographic Countermeasures. Electronics.

[25] Afdhal A., Muchallil S., Walidainy H., Yuhardian Q. Black Hole Attacks Analysis for AODV and AOMDV Routing Performance in VANETs. Proceedings of the International Conference on Electrical Engineering and Informatics (ICELTICs).

[26] Dhyani I., Goel N., Sharma G., Mallick B. (2017). A Reliable Tactic for Detecting Black Hole Attack in Vehicular Ad Hoc Networks. Advances in Computer and Computational Sciences.

[27] Grimaldo J., Martí R. Performance Comparison of Routing Protocols in VANETs under Black Hole Attack in Panama City. Proceedings of the International Conference on Electronics, Communications and Computers (CONIELECOMP).

[28] Purohit K.C., Dimri S.C., Jasola S. (2017). Mitigation and Performance Analysis of Routing Protocols Under Black-Hole Attack in Vehicular Ad-Hoc Network (VANET). Wirel. Personal Commun..

[29] Almutairi H., Chelloug S., Alqarni H., Aljaber R., Alshehri A., Alotaish D. (2014). A New Black Hole Detection Scheme for VANETs. Proceedings of the 6th International Conference on Management of Emergent Digital EcoSystems.

[30] Cherkaouia B., Beni-Hssanea A., Erritali M. Quality Control Chart for Detecting the Black Hole Attack in Vehicular Ad-Hoc Networks. Proceedings of the 8th International Conference on Emerging Ubiquitous Systems and Pervasive Networks (EUSPN 2017).

[31] Rawat D.B., Bista B.B., Yan G. Securing Vehicular Ad-Hoc Networks from Data Falsification Attacks. Proceedings of the IEEE Region 10 Conference (TENCON).

[32] Leinmuller T., Schmidt R.K., Schoch E., Held A., Schafer G. Modeling Roadside Attacker Behavior in VANETs. Proceedings of the IEEE Globecom Workshops.

[33] Grover J., Laxmi V., Gaur M.S. (2013). Attack Models and Infrastructure Supported Detection Mechanisms for Position Forging Attacks in Vehicular Ad Hoc Networks. CSI Trans. ICT.

[34] Nayak G.N., Samaddar S.G. Different Flavours of Man-In-The-Middle Attack, Consequences and Feasible Solutions. Proceedings of the 3rd International Conference on Computer Science and Information Technology.

[35] Ahmad F., Franqueira V.N.L., Adnane A. (2018). TEAM: A Trust Evaluation and Management Framework in Context-Enabled Vehicular Ad-Hoc Networks. IEEE Access.

[36] European Telecommunications Standards Institute (2011). Intelligent Transport Systems (ITS); Vehicular Communications; Basic Set of Applications; Part 2: Specification of Cooperative Awareness Basic Service, ETSI TS 102 637-2.

[37] Santa J., Pereñíguez F., Moragón A., Skarmeta A.F. (2013). Vehicle-to-Infrastructure Messaging Proposal Based on CAM/DENM Specifications. Proceedings of the IFIP Wireless Days (WD).

[38] Tobin J., Thorpe C., Murphy L. An Approach to Mitigate Black Hole Attacks on Vehicular Wireless Networks. Proceedings of the IEEE 85th Vehicular Technology Conference (VTC Spring).

[39] VEINS Vehicles in Network Simulation, The Open Source Vehicular Simulation Framework. http://veins.car2x.org.

[40] Sommer C., German R., Dressler F. (2011). Bidirectionally Coupled Network and Road Traffic Simulation for Improved IVC Analysis. IEEE Trans. Mobile Comput..

[41] SUMO Simulation of Urban MObility. http://sumo.dlr.de/wiki/Simulation_of_Urban_MObility.

[42] OMNET OMNET++: Discrete Event Simulator. https://omnetpp.org/.

[43] Wegener A., Piórkowski M., Raya M., Hellbrück H., Fischer S., Hubaux J.P. (2008). TraCI: An Interface for Coupling Road Traffic and Network Simulators. Proceedings of the 11th Communications and Networking Simulation Symposium.

[44] Alishev D., Hussain R., Nawaz W., Lee J. Social-Aware Bootstrapping and Trust Establishing Mechanism for Vehicular Social Networks. Proceedings of the 85th Vehicular Technology Conference (VTC Spring).

[45] Chaqfeh M., Lakas A. (2016). A Novel Approach for Scalable Multi-hop Data Dissemination in Vehicular Ad Hoc Networks. Ad Hoc Netw..

